# Institutionalisation of convergent medical innovation: an empirical study of the MRI-guided linear accelerator in the Netherlands and the United States

**DOI:** 10.1080/14479338.2023.2213212

**Published:** 2023-05-15

**Authors:** Charisma Hehakaya, Ellen H. M. Moors

**Affiliations:** a Global Public Health & Bioethics, University Medical Center Utrecht, Utrecht, The Netherlands; b Innovation Studies, Copernicus Institute of Sustainable Development, Utrecht University, Utrecht, The Netherlands

**Keywords:** Institutionalisation, convergent innovation, convergent technology, convergence, complex innovation, organisational design

## Abstract

Although convergence is a major trend in the development of medical innovations, the implications of the institutionalisation of convergent innovation are understudied. This paper explores how the institutionalisation of convergent innovation affects the organisation of health care, by using operational domains and categories of the Non-adoption, Abandonment, Scale-up, Spread and Sustainability (NASSS) and the Institutional Readiness (IR) approach respectively. We use an illustrative comparative case study on the institutionalisation of MRI-guided linear accelerator (MR-Linac) technology in the Netherlands and the United States. Empirically, we conducted 66 interviews with different professionals in the health care system around MR-Linac. The findings show that institutionalisation of convergent innovation affects the organisation of health care by: changing the traditional organisation of solving a medical problem, thereby transforming and reorganising work in the health care environment, providing opportunities for individual user development, collective action and cross-sectoral developments, and requiring the additional work of evaluating convergent innovation, including administrative tasks, innovation and research activities within and across institutions. The insights offered are also relevant for understanding convergence in the medical field, and for rethinking medical innovation in general.

## Introduction

The increasing convergence of knowledge, competencies and tools from different fields has led to the emergence and diffusion of health care-related innovations worldwide, such as biotechnology, personalised medicine, nanotechnology and information technology (Roco, 2003; Shmulewitz et al., 2006; Venkatesan, 2010). This so-called convergent innovation has been defined ‘as a solution-oriented approach that combines technological and social innovation in a form of “meta-innovation” that integrates human and economic development outcomes through behavioural and ecosystem transformation’ (Dubé et al., 2014, p. 125). The convergence of knowledge, competences, and tools from different scientific disciplines and domains in a single process or product in health care, then results in a new supporting method for disease prevention, diagnosis, monitoring or therapy (Chen et al., 2015; Curran & Leker, 2011; National Research Council 2014, 2014; Roco, 2003).

Digitalisation, an illustrative converging trend in health care (e.g., artificial intelligence, machine learning and deep learning), is often used to support users’ decision-making, and frequently requires and provides multidisciplinary knowledge (Bukowski et al., 2020; Dougherty & Dunne, 2012). This enables disruptive work approaches, regulations and functions for users and organisations, including the potential for cost savings and creativity (Bezemer et al., 2019; Boyd & Holton, 2018; Shmulewitz et al., 2006; Sraml Gonzalez & Gulbrandsen, 2021; Yu & Kohane, 2019). Digital convergence may therefore adapt both the delivery and the financial component of health related services, dealing with a wide variety of mechanisms used to fund, commission and deliver the service in question (Connelly & Fiorentini, 2021).

Beyond the clinical and technical novelties, convergent innovations can create new forms of care, with changes to the organisation, delivery and working environment of the entire health care system. Institutionalisation of convergent innovation thus focuses more on the process of innovating through systemic, social and behavioural changes rather than solely on technology development (Dubé et al., 2014; Rikkiev & Mäkinen, 2013; Venkatesan, 2010). We define institutionalisation as ‘the process to implement an intervention into all subsystems of an organisation in its institutional context with all socio-cultural, economic, political and regulatory influences present’ (Hargrave & Ven, 2006; Lounsbury & Crumley, 2007; Stephen & Barley, 1997). Convergent innovations are changing the way traditional health care products and services are developed, produced and delivered. These innovations integrate the formerly distinct sectoral boundaries of medical interventions, knowledge and actors, and target multiple interacting components, several dimensions of complexity, and aspects of uncertainty and organisation (Phillips et al., 2017; Seward et al., 2021).

The assimilation of traditionally distinct technical and social features, value networks, and organisation aspects in convergent innovation creates additional institutional complexity and uncertainty (Lounsbury & Crumley, 2007; Moors et al., 2018; Phillips et al., 2017). Institutionalising convergent innovation may therefore bring opportunities, but also challenges, since health care systems deal with traditionally separate actors with specific roles, strong routine practices, techniques and strict jurisdictions and regulatory practices (Moors et al., 2018).

There is no doubt that convergent innovations have an important role in changing and developing health care and beyond. The literature so far has mainly focused on describing convergent innovation rather than explaining its implications for the institutional context (Phillips et al., 2017; Shmulewitz et al., 2006). Institutional effects may play an important role in the dynamics of convergent innovation, but these effects have received little or no attention (Coenen & Truffer, 2012). As such, exploring how the institutionalisation of convergent innovation affects the organisation of health care is relevant.

We use the analytical frameworks from the Non-adoption, Abandonment, Scale-up, Spread and Sustainability (NASSS) (Greenhalgh et al., 2017) and Institutional Readiness (IR) (Webster & Gardner, 2019) to explore how the institutionalisation of convergent innovation affects the organisation of health care. The NASSS framework focuses on the factors affecting the implementation processes of medical innovations. The IR framework emphasises how technological innovations are diffused in institutional structures within a particular health care context, thereby exploring the necessary work done by involved stakeholders. The NASSS framework describes key elements in the institutionalisation environment, while the IR framework facilitates the understanding of the institutional readiness of implementing convergent innovation. In addition, the IR framework adds a more systemic perspective on the interaction between readiness of organisations and the institutional context.

By using a combination of the NASSS and IR framework, we go beyond the more traditional studies on determinants of adoption focusing mainly on barriers to innovation. We bring in a more dynamic perspective, in which we move away from seeing technological innovation as a stable object or process, but rather as being continual reconfigured by enactment of specific enablers, processes of evaluation, and organisational receptivity. In this way, we integrate and build on recent work from Innovation Studies and Science and Technology Studies.

We focus on the institutionalisation of the 1.5T MRI-guided linear accelerator (MR-Linac) technology as an illustrative case study of a convergent innovation. The MR-Linac integrates two traditionally separate modalities, diagnostic MRI guidance and radiation therapy, into one device for cancer treatment. Together with digitisation in the MR-Linac technology, it requires different knowledge bases and competences of involved actors.

## Theoretical framework

Health care related domains are currently transforming and recombining in many novel ways, with convergence of disciplines and sectors, and convergence of ideas and expertise with multiple actors involved. The involvement of multiple actors and disciplines means that convergent innovations in health care cannot be understood without a better understanding of the institutional practices including the redistributed knowledge, responsibilities, agency and power (Lounsbury & Crumley, 2007; Moors et al., 2018). To illustrate, traditional health care providers have to redefine their jobs and knowledge, and new health care professions and adapted regulations are needed. In other words, convergent innovations need to be legitimised, aligned and institutionalised in new categories, routines, norms and regulations (Battilana et al. 2017; Faulkner & Poort 2017).

According to Dubé et al. (2014), the essential building blocks of convergent innovation include: (1) a new technology-based invention, (2) developments in the way individuals interact, which opens up new opportunities to individuals as well as the community in which they interact, (3) intra- and interorganisational development that facilitate new activities, (4) advancement in financial instruments and payment systems, and (5) a cross-sector collective action in which institutions are modified or created. Accordingly, convergent innovation manifests through collaborative innovating, including the knowledge available from individuals in terms of rational and less rational motives for engagement and communication (Dubé et al., 2014). The collaborative nature of convergent innovation may encounter difficulties in the siloed health care environment with strict regulations, work routines, values, attitudes, competencies among the individuals and organisations involved, their interactions and the institutional context.

In general, individual, organisational, and contextual elements appear to be important predictors of how hospitals bring new technology into the organisation (Kimberly & Evanisko, 1981). This process may be assumed more complex given the collaborative nature of convergent innovation. Understanding the key elements of institutionalisation of convergent innovation could then help in exploring its impact on the organisation of health care.

The Non-adoption, Abandonment, Scale-up, Spread and Sustainability (NASSS) framework (Greenhalgh et al., 2017) describes key elements in implementation of innovation. This framework was applied to several health-related case studies, also including convergent innovations in health care (Benson, 2019; Dijkstra et al., 2019; Edridge et al., 2020; Hehakaya et al., 2020; Papoutsi et al., 2020). Empirical applications ranged from service-oriented innovation to more product-oriented innovations, such as e-health interventions and digital technologies. The analytical framework focuses on implementing and sustaining a new medical innovation over time, according to seven interacting domains (Greenhalgh et al., 2017):

- *the clinical condition* to be treated, addressing clinical aspects, comorbidities and sociocultural aspects
- *the technology* to be adopted, including material and technical aspects, compulsory knowledge and knowledge generated by technology and its supply model
- *the value proposition*, describing worth for the patient, provider, payer and society at large
- *the adopter system*, defining the changes in roles, practices and identities of staff, as well as the effect on patients and the extended network of lay caregivers
- *the organisation*, providing the technology, reflecting on its capacity, current practices and routines, readiness and the work necessary for implementation
- *the wider institutional and social context*, describing health and fiscal policy, as well as professional and regulatory bodies
- *the organisational resilience and technological development over time* as the above domains interacts and evolve dynamically in an institutional setting.

The NASSS framework can also be used to examine the degree of complexity in the domains described above: simple (few components, predictable), complicated (multiple interacting components, still largely predictable) or complex (dynamic and unpredictable). Innovations characterised by multiple complex domains rarely get successfully adopted, scaled up, spread and sustained (Greenhalgh et al., 2017). As such, the NASSS framework could also define degrees of complexity in the environment of the innovation and could forecast the success of an innovation.

We use an additional framework to identify challenges of convergent innovations at the organisational and the systemic level regarding the interaction between readiness of organisations and the institutional context. The Institutional Readiness (IR) framework (Webster & Gardner, 2019) increases understanding of the institutional readiness of implementing convergent innovation in more depth. Health care contexts are characterised by considerable complexity and heterogeneity including different stakeholders (Bonastre et al., 2007). This explains why the inter- and intra-organisational networks around convergent innovations regularly integrate a wide variety of practices, cultures and routines from often traditionally distinct disciplines, institutions and sectors (Dubé et al., 2014; Jha et al., 2014; Pope et al., 2013). The IR perspective adds insights into the organisational dynamics and challenges for new technologies at a systemic level, and specifically when and how a particular organisation is institutionally ready for a new technology. Its operational categories focus on how new technologies are engaged with and made sense of through the cultural processes and institutional structures within and outside of a specific organisation.

In particular the following institutional readiness categories, *(e)valuation processes in place*, *enactment through specific enablers*, and *receptivity*, add complementary insights to the NASSS framework regarding the institutional readiness of convergent innovation:

- The category *(e)valuation processes in place* is relevant in encouraging appropriate (national) protocols and guidelines, and the (shared) assessment of the value of new technologies in specific health care settings. Evaluation guidelines are also relevant to both the context and the guidance function of research, which affects the evaluation processes (Moors et al., 2018). For instance, the institutionalisation of medical technology is more stringent in the European health care setting compared to the US; e.g., recommending that evaluation processes embed cost-effective innovations into European health care, and to discontinue those interventions that have little or no added value (Burton et al., 2019; Helfrich et al., 2019; Moes et al., 2019; Verkerk et al., 2018). This category therefore complements the value proposition domain in order to prosper in the wider institutional and social context. Evaluation processes can also add insights into important enablers for institutionalising convergent innovation into the hospital environment, such as the needed medical protocols and guidelines.
- *Enactment through specific enablers* stresses how a variety of actors with different roles are represented in the adoption of the convergent technology. This category increases insights into the roles and tasks of specific facilitator stakeholders in the adopter system, the organisation and in the wider institutional and social context. Moreover, this category can also provide insights into specific private actors who are perceived as important for the development of convergent innovation (Dubé et al., 2014).
- *Receptivity* considers the changes and structures in an organisation in anticipation of unexpected events and challenges when implementing the convergent innovation in the wider institutional and social context. This category offers specific insights of responsiveness to the organisation, into, for instance, the external efforts to create legitimacy and to counter potential resistance in the wider institutional and social context. This category enables a better understanding of the actions an institution undertakes to institutionalise convergent innovation (Dubé et al., 2014).

Summarizing, complementing the NASSS framework with the discussed IR categories can provide useful insights into how convergent innovation affects the organisation of health care. A detailed analysis of the NASSS’ domains provides insights into key elements in the institutionalisation environment for convergent innovation, while the mentioned institutional readiness categories add a further systemic level perspective on the interaction between readiness of organisations and the wider institutional and social context.

## Methodology

### Research design and illustrative case study

We used an exploratory qualitative research method to explore how the institutionalisation of convergent innovation affects the organisation of health care. The analytical frameworks act as a starting point for the identification of important elements in institutionalising convergent innovation. We use the institutionalisation of the 1.5T MRI-guided linear accelerator (MR-Linac) technology in the Netherlands and the US as an illustrative comparative case study of convergent innovation.

The MR-Linac is currently being introduced in hospitals worldwide (Adam & Kenny, 2015; Murray & Tree, 2019; Mutic & Dempsey, 2014), and reflects different layers of convergent innovation, integrating different knowledge bases. It integrates the fields of high-precision cancer diagnosis and non-invasive, real-time radiotherapy in one device, including ongoing software development (Raaymakers et al., 2009). MR-Linac also includes ongoing software developments to enable automation processes during treatment, and to collect real-time anatomical and functional information about the tumour, and the organs at risk (Hall et al., 2019; Kiser et al., 2019). The involvement of diagnostic MRI in radiotherapy is also seen to transform ways of working, either within or outside the radiation oncology department (Hall et al., 2019). MR-Linac enables opportunities for professional development and new knowledge networks between actors, such as between physicians from radiotherapy and radiology as well as information technologists. This may increase innovation processes including the development of new treatment pathways and the regulations, guidelines, norms and new organisational responses involved. It may also affect the traditional capacity for oncology care and create new treatment avenues to meet public needs. Nevertheless, the institutional effects of MR-Linac on the delivery and organisation of health care are still understudied (Hehakaya et al., 2020).

We used interview data from our earlier empirical studies reporting on the opportunities and challenges of introducing MR-Linac in the Dutch and US health care systems (Hehakaya et al., 2020, 2022). These studies are published in Frontiers in Oncology, and Advances in Radiation Oncology clinical journals, respectively. In this study, we used these raw interview data for another research purpose to explore how the institutionalisation of convergent technology, MR-Linac, affects the organisation of health care in two different geographical health care settings, i.e., in the Netherlands and the US. It is useful to study such different health care settings to identify country specific similarities and differences regarding the involved stakeholders, organisational procedures, dominant existing routines, professional identities, and legal and regulatory standards (Caccia-Bava et al., 2006; Greenhalgh et al., 2017; Lettieri & Masella, 2009; Pope et al., 2013).

The Netherlands was the first country to introduce and implement the 1.5T MR-Linac. The University Medical Centre Utrecht (Utrecht, The Netherlands) developed this convergent innovation in collaboration with its manufacturer, Elekta AB (Stockholm, Sweden) (Kerkmeijer et al., 2016; Lagendijk et al., 2014). Multiple MR-Linac devices have now been installed worldwide, and especially in the US (de Mol van Otterloo et al., 2020). The study of the institutionalisation of MR-Linac in the Netherlands is insightful because of its early adopter experience, its extensive experience with technology institutionalisation, compared to its institutionalisation in the US, which included later adopter experience. Furthermore, the study of the US health care system, a more privatised than the more public-financed health care systems in Europe such as in the Netherlands, is also relevant to study possible differences in implementation processes of convergent innovation (Clancy & Cronin, 2005; Lievens et al., 2020).

### Data collection

We conducted interviews with different stakeholders in the two health care settings. Users who directly operate the MR-Linac technology, such as radiation oncologists, (clinical) physicists and radiation technologists were interviewed. Furthermore, we included MR-Linac developers, manufacturer representatives, health insurers, ICT staff, referring physicians, radiologists and nuclear medicine physicians. The interview data was collected from more than one person per professional group, and included perspectives from both academic and non-academic, general hospitals in both health care settings in order to include variation in findings and to increase construct validity.

Forty-three interviews were undertaken in the Netherlands by one researcher between November 2018 and March 2019. This researcher also worked in the MR-Linac department at University Medical Centre Utrecht in the Netherlands. Interviewees were included from five academic and three non-academic Dutch hospitals, of which two hospitals were early MR-Linac adopters. In the US, 23 interviews in five academic and two non-academic hospitals were undertaken by two researchers in 2020. One of these researchers also worked as a radiation oncologist at a hospital providing MR-Linac treatment in the US. Interviewees were mostly identified from a single US hospital involved in the early implementation of MR-Linac. Appendix A provides a detailed overview of the respondents and their roles and responsibilities related to MR-Linac.

### Data analysis

We followed an abductive research approach (Kovács et al., 2005). This involved coding transcripts both inductively (starting from empirical data) and deductively (iteratively comparing preliminary results with concepts derived from theory). In order to explore how convergent innovation affects the organisation of health care in both the Netherlands and the US, we analysed the interview transcripts for similar and distinct findings about implementing MR-Linac sequentially according to the NASSS domains and the IR categories, using NVivo© software. In a first round, we applied axial coding, systematically identifying areas of interest based on the NASSS domains and IR categories. This involved an iterative process with multiple rounds of coding. The codes developed were validated and discussed in team meetings. We triangulated responses across different respondents to identify consistent results. Finally, we reflected on how the convergent MR-Linac technology affects the organisation of health care in two different health care settings.

## Results

In order to explore how the institutionalisation of convergent innovation affects the organisation of health care, we analysed MR-Linac implementation in the Netherlands and the US. We found similarities and differences when implementing MR-Linac across both countries. We report the main findings according to the NASSS domains and IR categories.

### Clinical condition

The first domain addresses the clinical, comorbidities and sociocultural aspects of the condition to be treated. We identified a strong homogeneity in the clinical condition in MR-Linac implementation across the Dutch and US cases. Treatment on MR-Linac potentially reduces toxicity, improves tumour control and implies fewer hospital visits and less hospitalisation for patients. This improves patient convenience and quality of life. However, there is still a lack of evidence regarding clinical effectiveness for specific tumour indications, including knowledge about which patients benefit most from treatment on MR-Linac.

### Technology

This domain addresses requirements of the material and technical features of the technology. Similar technology features, knowledge and infrastructure were found when implementing MR-Linac in the Netherlands and the US. Real-time superior MRI guidance during treatment allows more targeted radiotherapy and the collection of anatomical and functional information about the tumour, which leads to additional understanding of individual treatment response and personalised treatment avenues. MR-Linac implementation requires substantial organisational and technical investments, due to the combined functionality of both the diagnostic MRI device and the radiation delivery. To illustrate, a MR-Linac device costs 10 million euros without the necessary infrastructure, such as imaging compatibility, safety, and its accompanying software development, quality assurance and applicable protocols. Furthermore, individual technology users must not only possess knowledge of radiotherapy and MRI, but also must remain informed about the technology’s software developments. This requires ongoing learning and active input about operating MR-Linac by technology users, which may result in inter- and intra-variability in treatment procedures.

### Value proposition

This domain considers whether the technology is valuable to implement and for whom it generates value. The value proposition of implementing MR-Linac shows similar clinical and technological aspects in both the Dutch and the US case, and also professional, organisational and economic expectations. Technology users would implement (1) a state-of-the-art evolving technology in the field of radiation oncology with opportunities, for (2) improved quality treatment, (3) in-hospital efficiencies, (4) research and funding possibilities, (5) competence enhancement and technical expertise, and (6) hospital profiling with increased demand for care. For instance, the absence of pre-treatment planning within an MR-only online workflow and the combination with diagnostics during the course of radiotherapy decreases reliance on the radiology department, leading to several efficiencies. The automation of tasks within MR-Linac workflow is perceived to allow faster treatment, which reduces treatment times for patients and providers.

Conversely, the final value proposition of MR-Linac is still uncertain, as there is a lack of empirical evidence for short- and long-term treatment outcomes. This also creates scepticism among treatment providers regarding MR-Linac’s added-value for tumour indications that already got effective treatments. The clinical added-value of MR-Linac, such as cost-effectiveness, will vary across countries due to national treatment standards, tumour indications and patient populations.

### Adopter system

This domain addresses the adoption of the technology by staff, patients and the extended network of lay caregivers. We found both comparable and distinct findings in the adopter system for implementing MR-Linac in the Netherlands and the US respectively. The acquisition of MRI knowledge and competences would change the traditional roles, practices and identities of radiation oncologists, technologists and physicists. Especially the autonomy and involvement in the treatment workflow of radiation technologists would particularly increase. Implementing MR-Linac also requires increased interdisciplinary cooperation between radiation oncologists, radiologists, imaging professionals and ICT experts, due to the need for MRI knowledge and competences during treatment and ongoing software developments. In both the Netherlands and the US, interviewees mentioned a lack of MRI competences among radiation oncologists and technologists.

In the Netherlands, interviewees reported the need for cooperation with the referring physician during implementation of MR-Linac to ensure treatment access, as well as with health care insurers to ensure development of appropriate reimbursement schemes. The lack of evidence about effectiveness is perceived to result in ethical discussions between physicians and payers about delivering unproven treatment. The limited availability of radiation technologists in the Netherlands is another concern when implementing MR-Linac.

Interviewees in US addressed the existence of patient groups from disadvantaged socioeconomic backgrounds, which could limit the access to new, costly treatment such as MR-Linac. The interviewees also indicated that well-insured patients might believe that high-quality hospitals offer state-of-the-art treatment, while more marginalised communities (e.g., the African-American community) might be more reluctant to accept new therapy.

### Organisation

This domain relates to the organisation’s capacity, current routines and work necessary for implementation of the technology. We found both comparable and distinct findings in the organisation domain. In both countries, radiotherapy centres often lack the required combination of MR-imaging and radiation facilities. This results in substantial investments for implementing MR-Linac, including technology costs and necessary changes to hospital infrastructures. A long-term structural investment in team work, professional learning and training is also needed in order to use the MR-Linac technology effectively, both now and in the future, given the present ongoing technology development. Changes in the delivery of care and the reallocation of tasks and responsibilities also require adaptations in clinical practice guidelines and personnel policies.

Also differences in MR-Linac implementation appear between the Netherlands and the US. The Dutch interviewees elaborated on the necessary cooperation with competitive physicians outside the radiation oncology department to ensure treatment access. The Dutch interviewees also reported potentially conservative behaviour and resistance among technology users as a response to delegating tasks and changing daily routines. In the US, the interviewees noted that the Covid pandemic was an important factor in that it had led to changes in resource allocation, such as committing more human resources to primary care provision rather than to secondary tasks such as clinical evaluation of implementing MR-Linac.

### Wider institutional and social context

This domain addresses the wider institutional and socio-cultural context. Both comparable and distinct findings were found about the implementation of MR-Linac in the Dutch and US institutional and social context. There is a need to work with policy-makers to adjust clinical guidelines and formal licences to the redistribution of roles and tasks, such as for radiation technologists. There is also a need for better consensus regarding reimbursement fees among MR-Linac providers and health insurers.

Interviewees in the US case particularly noted that the external authorities for reimbursement focused more on safety issues than on demonstrating clinical effectiveness. In particular, the Dutch interviewees mentioned the pressure of academic publishing which constrains knowledge exchange and open communication about treatment implementation among technology users. This may hinder effective multicentre collaboration and knowledge exchange between hospitals implementing MR-Linac.

### The organisational resilience and technological development over time

This domain questions how much scope there is for adapting and coevolving the technology over time. MR-Linac’s development is still continuing, without any predefined endpoints. New software features are expected to affect decision-making in treatment delivery, work processes in the radiation oncology department, and outcomes for patients and providers (both individual health care workers and hospitals as a whole). Hence, the scope for adapting and embedding MR-Linac in hospitals is dynamic. These findings suggest that individual and organisational resilience is necessary in managing potential changes and unexpected developments in implementing MR-Linac.

### (E)valuation processes in place

This category identifies the necessary assessments of the outcomes of new technologies and how this is shared. It is broadly recognised in both the Netherlands and the US that evaluation processes need to be in place to evaluate treatment safety, outcomes and (cost)effectiveness. MR-Linac is being evaluated by, amongst others, the international MOMENTUM study, which is generating (cost)effectiveness evidence. Four European institutes, two institutes in the US, one in Canada, and the manufacturing industry for the MR-Linac (Elekta AB, Sweden) were involved in founding MOMENTUM (de Mol van Otterloo et al., 2020).

The Netherlands is a forerunner in evaluating MR-Linac. Evaluating clinical effectiveness for new medical technologies is perceived as being more important for care insurers, and in the new European Medical Device Regulation. In the US, interviewees are more motivated by federal regulators to demonstrate safety rather than effectiveness, to ensure reimbursement. This is perceived by all US interviewees, and the reason why clinical evaluation has not been widely integrated into the hospital department.

### Enactment through specific enablers

This domain identifies individuals or groups with the formal task to enable adoption of the technology and regulatory standards. It is necessary for specific technology users such as a radiation oncologist, radiation technologist and physicist, to engage with regulators, insurers and policy-makers in both countries. For instance, the implementation of new interventional procedures and personnel guidelines on MR-Linac technology requires endorsement from national regulatory authorities. Only Dutch interviewees indicated it was important for the Professional Association for Radiation Oncology (In Dutch: Nederlandse Vereniging voor Radiotherapie en Oncologie) to be engaged in the creation of appropriate staffing guidelines and referring physicians for treatment access.

In both cases, the manufacturer, Elekta, is important for enabling implementation by solving technical problems and translating research into technical specifications available for technology users (Kerkmeijer et al., 2016). The international MR-Linac Consortium, consisting of over 30 centres and the manufacturer, support technology development, knowledge dissemination and best practices at individual and institutional level (Kerkmeijer et al., 2016). This large scale academic-industrial partnership with all intellectual property rights and data sharing regulations needs to be recorded in a collaboration agreement.

### Receptivity

This domain emphasises the novel institutional structures in anticipation of unexpected events and challenges when implementing the new technology. In both the Netherlands and the US, implementing MR-Linac results in a high level of individual receptivity (e.g., individual workplace training for staff to acquire MRI skills and competences) and organisational receptivity (e.g., new developments in hospital infrastructure, interventional protocols, staffing guidelines and reimbursement fees). The MR-Linac consortium acts as an information and communication platform to expand knowledge on MR-Linac and prepare its implementation at individual and institutional level.

Table 1 summarises the similar and distinct findings regarding the institutionalisation of MR-Linac in the Netherlands and the US respectively. The first column in Table 1 addresses the NASSS domains and IR categories. The second and third columns address the similarities and differences in institutionalisation between the two health care settings respectively. The fourth column addresses illustrative quotes from some of the respondents.

**Table 1.** Similarities and differences in the institutionalisation of MR-Linac in the Netherlands and the United States.

NASSS domains/IR categories	Similarities in MR-Linac institutionalisation between the Netherlands and the United States	Differences in MR-Linac institutionalisation between the Netherlands and the United States	Illustrative quotes
Clinical condition	Potential for improved tumour control and reduced treatment toxicity Scope for treatment new clinical indications Lack of evidence of (cost)effectiveness		*‘The key feature is the ability to see the cancer, to see the target that we want to treat when we’re actually treating it to enable us to deliver the radiation dose more precisely. And it also offers the future potential to image the functional attributes of the target, which will further allow us to personalise the treatment by identifying patients that are responding to treatment and those that do not’*. (industry executive, US)
Technology to be adopted	Superior online image guidance during treatment Functional imaging of tumour and organs at risks Scope for new treatment strategies Substantial infrastructure, staffing and organisational requirements Continuous software development Technical complexities		‘*The treatment will be finished faster. We will reduce the patient’s burden if we reduce the 20 treatment fractions to 5. It can therefore save waiting lists. The present-day waiting list can last for about 6 weeks. Currently, patients are waiting additional hours for the final radiation plan’*. (radiation oncologist, NL) *‘I reckon MR-Linac is mainly affordable for large hospitals, due to capital intensive investments and highly skilled staffing. It’s an expensive device. It costs about three and a half times more than a conventional device’*. (radiation oncologist, US)
Value proposition	Advanced technology Potential for improved treatment quality Professional development opportunities In-hospital efficiencies Research and funding possibilities Lack of effectiveness evidence Uncertain return of investment	Cost-effectiveness potential of treatment can vary across health care systems	*‘MR-Linac is a very expensive device that costs millions. However, this can lead to massive savings as a result of low claims on surgical procedures and anaesthesia’*. (division manager, NL) *‘Hypofractionation allows prostate cancer treatment in one to three times instead of five to twenty times. That saves a lot of time. MR-Linac is an excellent device for this purpose’*. (radiation oncologist, NL)
Adopter system	New knowledge and competences Continuous learning curve Increased professional autonomy Increased interdisciplinary teamwork Redevelopment of tasks and responsibilities	NL: necessary collaboration with referral physician for treatment access NL: necessary cooperation with payer for reimbursement US: potential reluctance of patients towards expensive medical innovations	*‘I reflect about physics matters and the physicist about clinical matters. There is much more interaction between these two disciplines’*. (radiation oncologist, US) *‘It will create a more interesting and challenging job. I really enjoy working on new projects. I cannot think of working in an ordinary radiation therapy department’*. (radiation technologist, NL)
Organisation	Substantial infrastructure and organisational investments Changing staffing tasks and responsibilities Ongoing education and development programs Presence of professional silos	NL: necessary collaboration with referring departments for treatment access NL: conservative behaviour among healthcare professionals towards changes in clinical practice NL: ethical discussions among healthcare professionals to provide unproven treatment US: limited prioritisation of effectiveness evaluation	*‘Traditionally, there is a separation between the preparation and treatment on the conventional radiation system. The preparation includes the creation of a treatment plan based on CT. The next step is the treatment. These two steps are two separated practices. In addition, in most hospitals these are performed by different groups. They are often in other departments and the interaction between them is therefore limited. This separation disappears with MR-Linac. The treatment plan, the diagnostics, is also performed online on this device’*. (physicist, NL) *‘The radiotherapy department will require more competences with regard to MRI imaging. And how to interpret MRI images. There is also a need for the people actually at the machine to understand more about the dose symmetry of the treatment plan that’s delivered, which hasn’t been the case in the past. The doctor will need to increase their skills and the people actually operating the device will increase their skills. The physics group will need to increase their skills in terms of knowing more about MR imaging’*. (industry executive, US)
Wider institutional context	Necessary changes in traditional reimbursement structures Necessary changes in traditional staffing policy	NL: pressing publicity atmosphere between academic technology users to share implementation knowledge	*‘I think it’s up to the individual organisations to come up with their own protocols and security measures’*. (radiation oncologist, US) *‘True innovation is best done outside of the confines of rules that are necessary within a governed society’*. (physicist, US)
Organisational resilience and development over time	Continuing technology development without clear endpoints Uncertain clinical, economic, and organisational results over time		*’MR-Linac is a highly complex innovation. Practitioners should have experience to use the device correctly. MR-Linac is not a stable system. You have to be flexible and it’s not consistent with the conventional security of ensuring that practices go as planned’*. (physicist, NL) *‘The main internal challenge is the number of radiation technologists needed. This is simply a scarce resource. We continuously have a lot of vacancies for radiation technologists. We are not capable of dealing with this challenge’*. (radiation oncologist, US)
(E)valuation processes in place	Evaluation of technology is necessary to evaluate treatment safety, outcomes and (cost)effectiveness	US: very limited external incentive perceived to evaluate clinical effectiveness	*‘Comprehensive scientific research must have been conducted and reproduced in another hospital’*. (healthcare insurer, NL) *‘What would the cost be? What numbers are needed to actually make it a beneficial investment from a capital standpoint while the technological development still continues? I think that’s the main discussion’*. (radiation oncologist, US)
Enactment through specific enablers	Essential engagement between technology providers, policy-makers and insurers to set-up appropriate clinical practice guidelines and reimbursement The manufacturer is essential to translate research findings into technical specifications available for all hospitals	NL: preferred engagement with professional association to create appropriate clinical practice guidelines NL: essential collaboration with referring physicians for treatment access	*‘The radiation oncologist is the final decision-maker, but this could be more efficient. Currently, a radiation technologist is not allowed to approve a treatment plan’*. (division manager, NL)
Receptivity	Necessary workplace trainings to educate technology users International consortium to facilitate knowledge dissemination and best practices		*‘Especially in the early days, structured sessions where we lay out and define what success looks like and we foster an open environment for differing perspectives to be heard’*. (radiation oncologist, US) *‘Changing and creating a treatment plan online ad hoc requires a new attitude and mindset among many radiation oncologists. Many radiation oncologists use one day for the final treatment decision, which in this case is not possible when you have to decide right away’*. (radiation oncologist, NL)

NL = the Netherlands, US = the United States.

## Discussion

While convergence is a major trend in the development of medical innovations, studies of the implications for the organisation of health care are still scarce. We contribute by exploring how the institutionalisation of a convergent innovation, the MR-Linac technology, affects the organisation of health care. We explored and compared the institutionalisation of MR-Linac in the Netherlands and the US.

The findings show how the institutionalisation of convergent innovation affects the organisation of health care at technological (the integration of MRI and a linear accelerator into one device), individual (e.g., individual physicians in diagnostic radiology and oncology working together), organisational (e.g., engaging different departments and hospitals) and institutional levels (e.g., changing the regulatory environment). First, convergent innovation redefines and reorganises traditional working tasks of clinical users by tackling a compelling and complex health care problem. The convergence of diagnostic radiology and radiotherapy provides a real-time, improved understanding of individual treatment response, thereby enabling better-informed medical practice for both radiation oncologists and diagnostic radiologists. MR-Linac thus changes the traditional methods and delivery of radiation treatment and radiology into one common method. The realisation of these potential work changes, however, requires different efforts that enable an appropriate institutionalisation of MR-Linac. This resonates with the need for a high level of receptivity in the institutionalisation environment.

Secondly, convergent innovation empowers the development of new knowledge and competences for individual technology adopters. Technology users, such as radiation oncologists, technologists, and physicists have to learn and integrate fairly new knowledge (on MRI principles) and skills (on the use of advanced software in treatment) into their original knowledge base, i.e., the delivery of radiotherapy without MRI. Radiation oncologists have to acquire more institutional level knowledge (e.g., care insurance structures for new reimbursement schemes) and have be able to process technological and regulatory changes along with socio-cultural changes, such as changing responsibilities, working practices, personnel policies for new medical roles and a more collaboration-oriented hospital culture in general. Individual technology adopters need to further develop their competences in the technological, social, organisational field, and both deepen and broaden their knowledge of the convergent innovation at play.

Thirdly, convergent innovation fuels new means of intra- and interorganisational collaboration and emphasises the process of innovating and shared sense-making. MR-Linac brings together physicians from radiation oncology, diagnostic radiology and in-house ICT experts. Convergent innovation thus unifies different knowledge flows into new working approaches to treatment, for example, by setting appropriate measurable outcomes for MRI and radiotherapy, and promoting more collaboration between distinct hospital departments. This increases internal hospital working practices, new guidelines, communication and research for individual technology users (Jacobs et al., 2021; Lievens, 2017). It also leads to additional administrative requests in the providing organisation, such as changes in patient allocation across diagnostic radiology and radiation oncology departments and adaptations in financial and payment structures.

Fourthly, convergent innovation increases interaction and collective action between individual technology users from different backgrounds rather than solely technological development. Convergent innovation integrates intellectually-diverse actors and traditionally separated institutions (e.g., hospitals, the manufacturing industry and health policy-makers), which changes the traditional hospital organisation and fosters novel work-related interactions. The actors involved in convergent innovation must find a way to collaborate and understand how their particular knowledge and competences contribute to institutionalisation. For instance, MR-Linac’s ongoing software development requires collaboration between individuals with clinical, technical and ICT knowledge (Hall et al., 2019; Ibbott, 2018; Tree et al., 2018). Individual adopters implementing convergent innovation have to engage with actors beyond their daily work routines in the institutional health care setting.

Fifth, evaluation processes are gaining more attention in the institutionalisation of convergent innovation, in terms of the demonstrating treatment outcomes, adapted clinical guidelines and reimbursement opportunities and uncertainties in the wider institutional context. For instance, MR-Linac’s treatment outcomes still need to be determined, which creates uncertainties for patients, providers and health care insurers (Aarons et al., 2012; Hall et al., 2019). Moreover, convergent innovation may be attractive to health care workers to acquire new knowledge and skills, obtain increased autonomy, and further develop their tasks and responsibilities. Evaluation processes are necessary to identify these changes, which need to be included in formal guidelines such as clinical practice and personnel policies.

MR-Linac’s ongoing digital development also requires evaluation, which is a continuous activity in response to internal and external decisions or needs of stakeholders. This resonates with the digitisation trend in medical innovation, which fosters personalised medicine and requires case-by-case regulation and policy adaptation (Deans et al., 2018; Kukk et al., 2016; Shah et al., 2019; Shaw et al., 2019). This also corresponds to the need for knowledge networks, and the development of clinical trials, treatment development and regulatory structures, in the institutionalisation of medical practices (Rake et al., 2021; Thune & Gulbrandsen, 2017).

Sixth, convergent innovation creates an ecosystem for the organisation of health care, including the involvement of a wide variety of institutions. For instance, the evaluation processes of the MR-Linac is also supported by clinical and commercial technology users, and by incentives from regulators in the adopter system. Institutionalising MR-Linac shows the importance of enactment through specific enablers, such as health care insurers, policy-makers, professional associations and the manufacturer industry. This is important to unify different areas of expertise and encourage collaboration among these stakeholders, which would facilitate more evidence-based information on the use of convergent innovation. This corresponds with the needed receptivity and so-called innovation governance efforts, in which diverse public and private actors are involved, steering the stimulation and regulation of novel knowledge creation and innovation processes (Gifford et al., 2021) within the organisation, but also in a specific institutional health care setting.

The NASSS framework has proven to be useful for an overall understanding of the institutionalisation of MR-Linac, while the IR framework revealed key how MR-Linac is diffused in institutional structures, such as the role of a professional association, consortium and evaluation trials, for institutional readiness at a systemic level. Our findings also showed how NASSS domains are interdependent and interact in dynamic ways, thereby also providing insights into the complexity of the domains. For example, the value proposition of MR-Linac can be classified as highly complex because of the undefined cost-effectiveness potential and difficulties in formulating a reliable business plan. Hence, the clinical indication and technology domain of a convergent innovation such as MR-Linac can both be characterised as complex domains. The ongoing development of technology is expected to influence the work needed in the hospital and of the adopters, stressing a rather complex organisation and adoption system.

The NASSS framework claims that medical innovations with high levels of complexity in domains are rarely successfully adopted and sustained (Greenhalgh et al., 2017). Our empirical work, however, reveals that a high degree of complexity and uncertainty in multiple NASSS domains does not hinder the ongoing large-scale adoption of MR-Linac worldwide (de Mol van Otterloo et al., 2020). Proof of long-term adoption is yet to come, but convergent innovation with a high level of upfront investment and coordination may be difficult to remove from clinical practice. Evaluation processes are therefore necessary to promote and facilitate institutional readiness.

## Relevance and limitations

Our findings add to a better understanding of the organisation of health care in the future, in which convergent innovation and digitisation are expected to play an important role. To obtain an encompassing perspective on MR-Linac institutionalisation we included a broad range of stakeholders, including actors from both inside and outside the hospital environment. Applying the NASSS framework (Greenhalgh et al., 2017), complemented with complementary IR categories (Webster & Gardner, 2019), provided an in-depth understanding of the institutionalisation of convergent innovation on the organisation of health care both in the Dutch and the US health care setting. Future studies in other geographical health care settings, and studies of other types of convergent innovations, will add to the empirical evidence base of factors influencing the institutionalisation of convergent innovation (Cunningham & O’Reilly, 2018).

Limitations in the research design include the relatively small number of stakeholders interviewed in the US (23) compared to the Netherlands (43). Interviews in the US were also conducted during the Covid pandemic which may have affected respondent’s perspectives on how institutionalisation of convergent innovation affects organisation of health care. It was difficult to contact private and public insurance regulators in the US, whose insights regarding the regulatory and policy factors influencing institutionalisation of convergent innovation would have been valuable. Ethnographic research on the institutionalisation of convergent innovation could offer a fine-grained understanding of the behaviour and interactions of individual technology users within a particular health care context. Studying convergence in other clinical conditions to be treated and other technology domains could reveal more specific insights in institutionalisation (Gassmann et al., 2010).

## Conclusions

The institutionalisation of convergent innovations does affect the organisation of health care. By investigating MR-Linac institutionalisation in two different health care contexts, we presented a study in which convergent innovation changed the traditional professional practice of addressing a medical condition, and the work of individual actors and institutions involved. Convergent innovation transforms the traditional way in which a medical problem is solved, including changes in the work tasks and routines of technology users. Convergent innovation increases individual and collective knowledge and competences development, and innovation and research activities for engaged actors. This is ideally facilitated by the evaluation of convergent innovation on its overall outcomes, which requires collaboration between stakeholders within and across institutions. The insights offered are also relevant for understanding convergence in the medical field, and for rethinking medical innovations in general.

## Supplementary Material

Supplemental Material
