# Supplementary material for: Institutionalisation of convergent medical innovation: an empirical study of the MRI-guided linear accelerator in the Netherlands and the United States
Source: Innovation (North Syd). 2023 May 15;27(1):74–95. doi: 10.1080/14479338.2023.2213212 (PMC11809769; doi:10.1080/14479338.2023.2213212)
Supplement: Supplemental Material [file RIMP_A_2213212_SM8194.pdf]

Appendix A. Overview of interviewees in the Netherlands and United States

Overview of roles and affiliations of interviewees in the Netherlands. AMC = Academic medical center, MC = (non-academic) Medical center

Interviewee	Position	Seniority	Affiliation	Additional roles	Method	Duration (min)
R1	Computer scientist	Senior	AMC1	Research on MR-Linac	In Person	43
R2	Manager Imaging & Oncology dep.	Senior	AMC1		In Person	47
R3	Head of Imaging & Oncology dep.	Full Professor	AMC1	Research on functional imaging	In Person	44
R4	Insurance commissioner	Senior	AMC1		In Person	25
R5	Insurance commissioner	Senior	AMC1		In Person	31
R6	Member Board of Directors	Full Professor	AMC1	Research on open Science	In Person	30
R7	Member Board of Directors	Senior	AMC1		In Person	44
R8	Member Board of Directors	Full Professor	MC1	Radiologist	In Person	45
R9	Nuclear medicine physician	Full Professor	AMC1	Board Member National Education Committee Nuclear Medicine	In Person	43
R10	Nuclear medicine physician	Senior	AMC2		In Person	41
R11	Radiologist	Senior	AMC1		In Person	42
R12	Radiologist	Senior	AMC1		In Person	40
R13	Radiation oncologist	Senior	AMC1	Research on MR-Linac	In Person	37
R14	Radiation oncologist	Senior	AMC1	Research on MR-Linac	In Person	41
R15	Radiation oncologist	Senior	AMC1	Research on MR-Linac	In Person	43
R16	Radiation oncologist	Senior	AMC1	Research on MR-Linac	In Person	45
R17	Radiation oncologist	Full Professor	AMC2	Head of Radiation Oncology department, Research on MR-Linac,	In Person	46

				Board member European Society Radiotherapy & Oncology		
R18	Radiation oncologist	Senior	AMC3	Head of Radiation Oncology department	Telephone	39
R19	Radiation oncologist	Senior	AMC4		In Person	45
R20	Radiation oncologist	Full Professor	AMC4		In Person	48
R21	Radiation oncologist	Senior	MC2	Research on MR-Linac	In Person	41
R22	Radiotherapy technologist	Senior	AMC1	Research on MR-Linac	In Person	43
R23	Radiotherapy technologist	Senior	AMC1	Research on MR-Linac	In Person	39
R24	Radiotherapy technologist	Junior	AMC1		In Person	42
R25	Radiotherapy technologist	Senior	MC2		Telephone	46
R26	Radiotherapy technologist	Senior	AMC3		In Person	44
R27	Physicist	Full Professor	AMC1	Research on MR-Linac	In Person	53
R28	Physicist	Senior	AMC1	Research on MR-Linac	In Person	41
R29	Physicist	Full Professor	AMC1	Research on MR-Linac	In Person	45
R30	Physicist	Senior	AMC1	Research on MR-Linac	In Person	44
R31	Physicist	Senior	AMC2	Research on MR-Linac	Telephone	38
R32	Physicist	Full Professor	AMC2	Manager Radiation Oncology department	In Person	41
R33	Physicist	Full Professor	AMC3		Online	39
R34	Physicist	Full Professor	MC2	Research on MR-Linac	In Person	43
R35	Physicist	Senior	MC2	Research on MR-Linac	In Person	47
R36	Urologist	Senior	AMC1		In Person	31
R37	Urologist	Senior	MC3		Telephone	29
R38	Urologist	Senior	AMC4	Head of Urology department	Telephone	40

R39	Patient representative		National patient organisation		Telephone	31
R40	Health care insurer	Senior	Insurance company 1	Radiologist	Telephone	38
R41	Health care insurer	Senior	Insurance company 2		Telephone	42
R42	Managing Director	Senior	Manufacturing company		Online	46
R43	Managing Director	Senior	Manufacturing company		Online	33

Overview of roles and affiliations of interviewees in United States. AMC = Academic medical center, MC = (non-academic) Medical center

Interviewee	Position	Seniority	Affiliation	Additional roles	Method	Duration (min)
R1	Head of Imaging & Oncology dep.	Full Professor	AMC1	Research on functional imaging	In Person	44
R2	Radiotherapy technologist	Senior	AMC1	Research on MR-Linac	In Person	43
R3	Insurance commissioner	Senior	AMC1		In Person	46
R4	Head of IT for Oncology	Senior	AMC1	Research on functional imaging	In Person	44
R5	Medical oncologist	Senior	AMC1		In Person	41
R6	Nuclear medicine physician	Senior	AMC1		In Person	39
R7	Member Board of Directors	Senior	AMC1		In Person	44
R8	Member Board of Directors	Senior	AMC2		In Person	44
R9	Radiation oncologist	Senior	AMC3		In Person	39
R10	Radiation oncologist	Senior	AMC4		In Person	39
R11	Radiation oncologist	Senior	AMC2	Head of Radiation Oncology department, Research on MR-Linac	In Person	45
R12	Radiation oncologist	Senior	AMC2	Research on MR-Linac	In Person	53
R13	Radiation oncologist	Senior	AMC2	Research on MR-Linac	In Person	39
R14	Radiation oncologist	Senior	AMC5	Research on MR-Linac	Virtual	41

R15	Urologist	Senior	AMC4	Head of Urology department	In Person	40
R16	Urologist	Senior	MC1		In Person	29
R17	Radiotherapy technologist	Senior	AMC1		In Person	41
R18	Radiologist	Professor	AMC1		In Person	35
R19	Radiation oncologist	Senior	AMC4	Head of Radiation Oncology department	Virtual	39
R20	Radiation oncologist	Senior	AMC2	Research on MR-Linac	In Person	45
R21	Radiation oncologist	Full Professor	AMC2		In Person	48
R22	Radiation oncologist	Senior	MC2		In Person	41
R23	Market Access associate	Senior	Manufacturing company		Virtual	46
